# Hospital-acquired bloodstream infection: Indian perspective

**DOI:** 10.1186/cc11986

**Published:** 2013-03-19

**Authors:** R Agrawal, A Varma

**Affiliations:** 1 FEHI, New Delhi, India

## Introduction

This is a 1-year prospective study to determine the incidence, source and etiology of hospital-acquired bloodstream infection (HABSI) in the Indian context. The resistance pattern was also reviewed.

## Methods

A single-centre prospective study in a 35-bed ICU. HABSI was defined according to current CDC guidelines. HCAP, catheter-associated UTI (CAUTI) and skin-related infections causing BSI was also defined according to recent guidelines and analysed.

## Results

Out of 332 positive samples, 90 samples (*n *= 45) were HABSI. The microbiological analysis showed 60% were Gram-negative, 6% were candida and 27% were Gram-positive. The commonest isolate was klebsiella and MRSA was commonest in Gram-positive. The source of HABSI showed CRBSI was the commonest cause at 69%, which correlates with international data. Ventilator-associated pneumonia and CAUTI caused 9.5% BSI respectively. The resistance pattern among Gram-negative bacteria showed multidrug-resistant (MDR) and extreme drug-resistant (XDR) isolates were highest. See Tables 1 and 2.

**Table 1:** Source

Source	Total (%)
CRBSI	69
HCAP	9.5
CAUTI	9.5
Devices	7
Skin	5

**Table 2:** Resistance pattern

Resistance	Total (%)
Non-MDR	15
MDR	44
XDR	33
PDR	8

## Conclusion

The incidence of HABSI is 27%. Of this, CRBSI cause 70% and Gram-negative bacteria were commonest with high resistance. This is in contrast to western data where Gram-positive infections are common. Our study highlights need for stringent guidelines for CRBSI prevention.
